# LL37 Inhibits *Aspergillus fumigatus* Infection via Directly Binding to the Fungus and Preventing Excessive Inflammation

**DOI:** 10.3389/fimmu.2019.00283

**Published:** 2019-02-20

**Authors:** Xiao-Li Luo, Jian-Xiong Li, Hua-Rong Huang, Jie-Lin Duan, Ruo-Xuan Dai, Ru-Jia Tao, Ling Yang, Jia-yun Hou, Xin-Ming Jia, Jin-Fu Xu

**Affiliations:** ^1^Department of Respiratory and Critical Care Medicine, Shanghai Pulmonary Hospital, Tongji University School of Medicine, Shanghai, China; ^2^Clinical Translational Research Center, Shanghai Pulmonary Hospital, Tongji University School of Medicine, Shanghai, China; ^3^Zhongshan Hospital Institute of Clinical Science, Shanghai Institute of Clinical Bioinformatics, Zhongshan Hospital, Fudan University, Shanghai, China

**Keywords:** LL37, *Aspergillus fumigatus*, mycelium, inflammation, adhesion

## Abstract

The incidence of *Aspergillus fumigatus* infection and the rate of resistance to antifungal drugs have sharply increased in recent years. LL37 has been reported as a host defense peptide with broad-spectrum antibacterial activities. However, the role of LL37 during *A. fumigatus* infection remains unclear. Here, we examined the interaction between LL37 and *A. fumigatus* and found that synthetic LL37 could directly bind to the surface of *A. fumigatus*, disrupting the integrity of the cell wall *in vitro*. LL37 inhibited mycelial growth in a concentration-dependent manner, rather than fungicidal effect even at high concentration (e.g., 20 μM). Interestingly, low concentrations of LL37 (e.g., 4 μM) significantly attenuated mycelial adhesion and prevented the invasion and destruction of epithelial cells. Following LL37 treatment, the levels of proinflammatory cytokines released by *A. fumigatus*-stimulated macrophages decreased significantly, accompanied by downregulation of M1 type markers. In a mouse model of pulmonary *A. fumigatus* infection, LL37-treated mice showed lower amounts of fungi load, moderate pathological damage, and reduced proinflammatory cytokines. Further, LL37 transgenic mice (LL37+/+) were examined to investigate the effects of endogenous LL37 in an *A. fumigatus* infection model and showed lower susceptibility to *A. fumigatus* infection in comparison with wild-type mice. In addition, LL37 also played a protective role in an immunosuppressed mouse model of *A. fumigatus* infection. Thus, LL37 inhibits *A. fumigatus* infection via directly binding to mycelia and reducing excessive inflammation. LL37 or its analogs may therefore constitute potential drug components for *A. fumigatus* infection.

## Introduction

*Aspergillus fumigatus*, an opportunistic pathogen widely distributed in nature, is the leading cause of pulmonary Aspergillosis (1). Pulmonary Aspergillosis includes three subtypes—specifically, chronic pulmonary aspergillosis (CPA), allergic bronchopulmonary aspergillosis (ABPA) and invasive pulmonary aspergillosis (IPA)—which are associated with different immune statuses of susceptible hosts. The morbidity and mortality of IPA have sharply increased in recent decades due to a rise in immunocompromised individuals, such as patients receiving organ transplants or chemotherapy (2). Furthermore, the emergence of antifungal drug resistance limits the effectiveness of clinical treatment (3–5). Even worse, excessive inflammation and severe tissue damage can deteriorate a patient's condition and increase treatment difficulty (6).

LL37, a short fragment composed of 37 amino acids, is the unique member of human cathelicidin antimicrobial peptides (CAMPs). It presents with broad-spectrum antimicrobial activity against various pathogens, including prokaryotic, and fungal organisms (7, 8). Its positive charge allows it to bind to negatively charged phospholipid membranes of prokaryotic cells, prompting membrane penetration, the formation of transmembrane pores, and bacterial lysis (9). On the other hand, LL37 possesses diverse modulating properties on immune system such as the recruitment of inflammatory cells and the release of inflammatory factors, showing both proinflammatory and anti-inflammatory effects (10). LL37 is expressed in a variety of immune cells and epithelial cells. In different types of cells and tissues, LL37 has different physiological concentrations and often increases during infection.

Recently, a few studies have shown that LL37 expression is significantly upregulated in corneal epithelium and nasal tissue in response to *A. fumigatus* (11, 12), suggesting that LL37 may play an important role in *A. fumigatus* infection. Therefore, in the present investigation, we explored the possible effects of LL37 against *A. fumigatus* infection. We tested whether or not LL37 could bind to *A. fumigatus*, destroy cell wall structures and inhibit mycelium growth and adhesion *in vitro*. To test the immunomodulatory effects of LL37, the release of tumor necrosis factor alpha (TNF-α) and interleukin (IL-6) from *A. fumigatus*-stimulated bone marrow-derived macrophages (BMDMs) after LL37 treatment was evaluated. Subsequently, exogenous LL37-treated mice and LL37+/+ mice were involved, respectively to determine the *in vivo* effects of exogenous and endogenous LL37 on fungi clearance, pathological injury, neutrophil infiltration, and cytokine production during *A. fumigatus* infection. Overall, this study demonstrates that LL37 not only directly inhibits *A. fumigatus* hyphae growth and adhesion but also prevents *A. fumigatus*-induced excessive inflammation, thus providing new evidence for the dual therapeutic value of LL37 against *A. fumigatus* infection.

## Materials and Methods

### Animals

Specific pathogen-free C57BL/6 mice and FVB mice breeding pairs were purchased from the SLAC Laboratory Animal Center (Shanghai, China). LL37+/+ mice were produced via the microinjection of linearized plasmids expressing hCAP18/LL37 into fertilized eggs of mice bred with an FVB genetic background (Figures S1A,B in Supplementary Material). All of the mouse strains were housed in specific pathogen-free conditions within an animal care facility (Center of Laboratory Animal, Tongji University, Shanghai, China) until the day of sacrifice. All of the animal experiments were performed under the guidance and with approval from the Institutional Animal Care and Use Committee of Tongji University (Permit Number: TJLAC-015-002).

### Reagents

Human cathelicidin LL37 (LLGDFFRKSKEKIGKEFKRIVQRIKDFFRNLVPRTES) with a purity of 95% was purchased from Rockland Immunochemicals (Pottstown, PA, USA). Scrambled form sL37 (RSLEGTDRFPFVRLKNSRKLEFKDIKGIKREQFVKIL) with a purity of 95% was synthesized by GL Biochem (Shanghai, China). Anti-cathelicidin antibody was purchased from Abcam (Cambridge, UK).

### *A. fumigatus* Strains and Culture

Conidia (*A. fumigatus* train, Af293) harvest and growth into swollen conidia and hyphae were performed as described previously (13). Briefly, fungi were inoculated on Sabouraud Dextrose Agar slant and cultured at 37°C for 7 days. Conidia were collected with phosphate-buffered saline (PBS), filtered through a 40-μm nylon mesh, then stored at 4°C for use. To obtain swollen conidia (SC) and hyphae, resting conidia (RC) were incubated in Roswell Park Memorial Institute (RPMI)-1640 media at 37°C for 8 h to achieve swelling and for an additional 2 h to achieve germination.

### Cell Culture

BMDMs from mice were prepared as previously described (14). Bone marrow was extracted from the femur and tibia of 6–8-week-old female C57BL/6 mice. Cells were centrifuged following the removal of erythrocytes and then were differentiated into BMDMs in Dulbecco's Modified Eagle medium supplemented with 10% fetal bovine serum, 30% L929 supernatant, 1% antibiotic-antimycotic, and 0.1% β-Mercaptoethanol.

### Transmission Electron Microscopy (TEM)

*Aspergillus fumigatus* conidia were treated with 4 μM LL37 or sL37 and incubated in RIPM-1640 medium for 24 h. Subsequently, the mycelia were pelleted by centrifugation and prefixed in a solution of 5% glutaraldehyde in 0.1 M sodium cacodylate buffer for 2 h. Samples were then washed three times with 0.1 M sodium cacodylate buffer and postfixed with 1% osmium tetroxide for 3 h and were dehydrated with increasing concentrations of ethanol or acetone (i.e., 50, 70, 90, 100%), respectively. Following embedding and fixation, samples were cut into ultrathin sections using an ultramicrotome. After staining with uranyl acetate and lead citrate, the ultrathin sections were viewed under a JEOL JEM-1230 (80KV) transmission electron microscope.

### Hyphae Growth Inhibition Assay

*Aspergillus fumigatus* conidia were incubated in RPMI-1640 medium with different concentrations of LL37 or sL37 at 37°C for 12 h. Hyphae length was then measured under microscopy. Each group included at least 10 visual fields containing ≥50 hyphae.

### Adhesion Assay

Adhesion assay was performed as previous described (15). *A. fumigatus* conidia were incubated in a 96-well plate with RPMI-1640 media in the presence of different concentrations of LL37 or sL37 at 37°C for 24 h. The supernatant was removed and then the wells were washed three times with PBS. Adhesive capacity was estimated by staining the biofilms that had not been washed off with 0.5% crystal violet for 15 min. Then excess stain was washed with PBS for three times. Afterwards, the biofilms were decolorized with 95% ethanol. The density of the biofilms was analyzed by determining the absorbance of the decolorized solution at 570 nm. At the same time, the wells both before and after washing were photographed under a microscope.

### Epithelial Cell Damage Assay

A mouse alveolar epithelial cell line (MLE12) were plated on a 48-well plate in a monolayer formation and infected with *A. fumigatus* conidia in the presence of LL37 or sL37. Following incubation for 16 h, the supernatant was collected and transferred to a 96-well plate. The LDH released in the supernatant was detected using the CytoTox 96® non-radioactive cytotoxicity assay kit (Promega Corp., Madison, WI, USA) according to the manufactures' instructions. Then the corrected values in the following formula were used to compute percent cytotoxicity:

$$\text{Percent cytotoxicity = }100\times \frac{\text{Experimental LDH Release (OD }490\text{)}}{\text{Maximum LDH Release (OD }490\text{)}}$$

The Maximum LDH Release means a positive control (i.e., treatment with detergent).

### Binding of LL37 and *A. fumigatus* Analysis

*Aspergillus fumigatus* RC, SC or hyphae were incubated with or without 4 μM of LL37 dissolved in PBS for 30 min at room temperature. The samples were then washed three times with PBS and fixed in 4% paraformaldehyde for an hour before being blocked with 5% fetal bovine serum albumin (BSA) for an hour. Following washing with PBS for three times, samples were incubated with anti-LL37 antibody overnight at 4°C. Following washed with PBS for three times, samples were incubated with a FITC-labeled secondary antibody for an hour at room temperature. The RC samples were analyzed using a flow cytometer (Becton Dickinson, Franklin Lakes, NJ, USA) while SC and hyphae samples were visualized and imaged using a confocal microscope (Nikon Inc., Tokyo, Japan).

### Cytokines Secretion

The 3 × 10^5^ BMDMs were plated on a 48-well plate in a monolayer and infected with 1.5 × 10^6^ UV-killed *A. fumigatus* swell conidia or 3 × 10^5^ hyphae in the presence of different concentrations of LL37 or sL37. After stimulation for 16 h, the supernatant was collected. The concentration of TNF-α and IL-6 in the supernatant was detected using ELISA kits (eBioscience, San Diego, CA, USA) according to the manufactures' instructions.

### *In vitro* Killing Assay

Here, the 3 × 10^6^ BMDMs were plated on a 12-well plate in a monolayer and infected with 6 × 10^5^ viable *A. fumigatus* conidia in the presence or absence of 4 μM LL37 or sL37. After incubation for 6 h at 37°C, the fungi were scraped off and diluted with sterile PBS. Afterwards, SDA plates were inoculated with diluted solution and cultured at 37°C for 48 h. The killing capacity was estimated by counting the number of fungi colonies.

### Phagocytosis Assay

A phagocytosis assay was based on and developed as described previously(16). Briefly, *A. fumigatus* conidia were pretreated with a Fluoro Tag^TM^ FITC Conjugation Kit (Sigma-Aldrich, St. Louis, MO, USA) and peritoneal macrophages were isolated from C57/BL6 mice as described previously (17). Then peritoneal macrophages were cultured on a 12-well plate in a monolayer and each well was inoculated with FITC-labeled *A. fumigatus* conidia to achieve a multiplicity of infection (MOI) equal to 10. After 1 h of coincubation at 37°C with 5% CO_2_, the wells were washed with PBS before digested by 0.25% trypsin to collect the adherent cells. Then, the collected macrophages were labeled with anti-CD11b PerCP cy5.5 (M1/70) (eBioscience, San Diego, CA, USA) and samples were analyzed using a BD flow cytometer. The percentages of phagocytosis were calculated by the ratio of FITC^+^ macrophages to all macrophages.

### Murine Models of Pulmonary Aspergillus Infection

Mice were anesthetized by isoflurane inhalation, and then (2-10) × 10^6^ *A. fumigatus* conidia in 35 μl of PBS were instilled into the trachea by pressing tongue intratracheal instillation. For the immunosuppressed model, mice were administered with 40 mg/kg of the corticosteroid, triamcinolone acetonide (TargetMol, Boston, MA, USA) injected subcutaneously 1 day prior to infection, as previously described (18). At the indicated times, mice were killed and lung tissues were isolated for detection of CFU, inflammatory cells, and cytokines as well as histopathological analysis.

### Fungal Burden Analysis

Mice were killed at the indicated times after infection and lungs were dissected carefully, excised, and homogenized in PBS. For CFU, the homogenates were serially diluted and spread onto SDA plates. After incubated at 37°C overnight, the colonies were counted and normalized to lung weights.

### Histopathological Analysis

Lung tissues isolated from mice were fixed in 10% buffer formalin, dehydrated, and embedded in paraffin. Then the lungs were cut into sections and stained with hematoxylin & eosin (HE) or Gomori's methenamine silver stain (GMS) according to standard staining procedures at pathology platform of Servicebio Technology, Wuhan, China.

### Flow Cytometry Analysis

Lung tissues from mice were digested with collagenase to establish a single-cell suspension as described previously (13). Briefly, lungs were dissected and cut into very small pieces before incubated in collagenase digestion solution (10 mg collagenase type II in 10 ml PBS) for 1 h at 37°C. Next, the digested lungs were sieved through 40 mm sieve and treated with RBC lysis buffer. Then, cells were washed and stained with the following monoclonal antibodies: anti-mouse CD45-FITC (30-F11), anti-mouse Ly6G-BV421 (1A8), anti-mouse CD11b-PerCP cy5.5 (M1/70) (eBioscience, San Diego, CA, USA), and anti-mouse CD11c-APC (HL3), anti-mouse SiglecF-PE (E50-2440) (BD Biosciences, San Jose, CA, USA), after which the cells were analyzed by a flow cytometer (Becton Dickinson, Franklin Lakes, NJ, USA). Next, the data were analyzed with the Flow-Jo software: First, all of the cells were defined via FCS-A and SSA-A and then single cells were determined via SSC-A and FCS-W. Afterwards, we defined CD45^+^ cells as immune cells in lung tissue, from which Ly6G^+^CD11c^+^ cells were gated as neutrophils and SiglecF^+^CD11c^+^ cells were gated as macrophages, respectively (Figure S2 in Supplementary Material).

### RNA Extraction and Real-Time Quantitative RT-PCR

Total RNA was isolated using TRIzol reagent (Takara, Kusatsu, Japan). Reverse transcription was performed by utilizing a Primescript RT reagent kit (Takara, Kusatsu, Japan). Real-time quantitative PCR measurement was performed using SYBR Green reagent kit (Vazyme, Nanjing, China) as per the manufacturer's instructions, and the ABI 7500 sequence detection system. The amounts of transcript were normalized to GAPDH. Relative mRNA expression was plotted as fold changes calculated using the ΔΔCt method. The primers used for real-time RT-PCR were as follows: iNOS (forward: 5′- ACATCGACCCGTCCACAGTAT−3′; reverse: 5′- CAGAGGGGTAGGCTTGTCTC−3′); CXCL9 (forward: 5′- ATCTCCGTTCTTCAGTGTAGCAATG-3′; reverse: 5′- ACAAATCCCTCAAAGACCTCAAACAG-3′); CXCL10 (forward: 5′- AGGGGAGTGATGGAGAGAGG-3′; reverse: 5′- TGAAAGCGTTTAGCCAAAAAAGG-3′); GAPDH (forward: 5′- AGGTCGGTGTGAACGGATTTG-3′; reverse: 5′- TGTAGACCATGTAGTTGAGGTCA-3′).

### Statistical Analysis

Statistical analysis was performed using GraphPad Prim 5.0 software. The graph represents at least three dependent experiments and data were presented as mean ± Standard Deviation (SD). Log-rank testing was used to evaluate the equality of survival curves. The other statistical differences were determined by the two-tailed unpaired *t-*test and one-way ANOVA. It was considered statistically significant when *p* < 0.05.

## Results

### LL37 Binds Directly to *A. fumigatus* and Destroys Cell Wall Integrity

**Figure 1:**
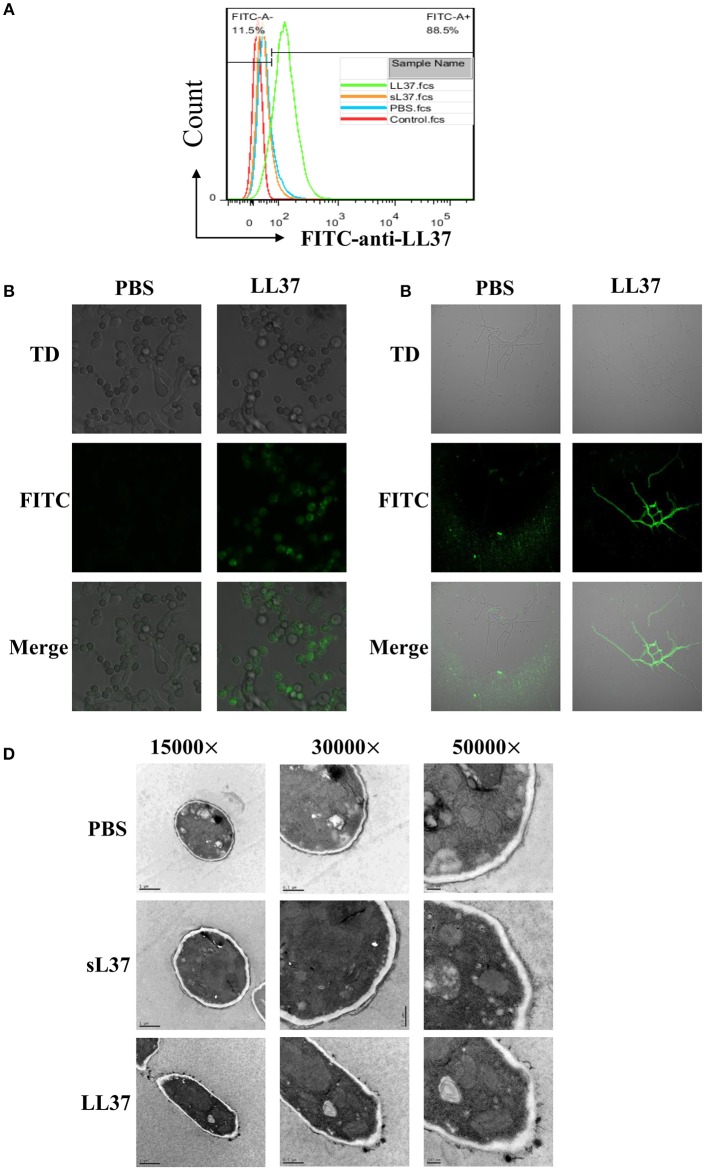
The direct interaction between LL37 and *A. fumigatus. A. fumigatus* resting conidia, swell conidia and hyphae were incubated with or without LL37 dissolved in PBS at room temperature for 30 min. After being washed and fixed, samples were stained with anti-LL37 antibody and FITC-labeled secondary antibody. Resting conidia **(A)** was analyzed by flow cytometer. Swell conidia **(B)** and hyphae **(C)** were visualized and imaged using a Nikon confocal microscope. **(D)** *A. fumigatus* conidia were incubated with LL37, sL37, or PBS for 24 h to grow into hyphae state. Following fixation and embedding, samples were cut into ultrathin sections and analyzed by transmission electron microscope. The data are representative of three independent experiments. PBS, phosphate buffer saline.

To investigate the direct antimicrobial activity of LL37 against *A. fumigatus*, we initially performed immunofluorescence staining of LL37. The direct binding of LL37 to the surface of *A. fumigatus* resting conidia was proved by flow cytometer after 30 min of coincubation, as well as swell conidia and hyphae observed under confocal microscopy (Figures 1A–C). Furthermore, to assess the effect of LL37 on the structure of *A. fumigatus*, mycelia grown from conidia in the presence of 4 μM LL37 were visualized by transmission electron microscope. The images revealed obvious alterations of cell wall integrity prompted by LL37—that is, the cell walls were more likely to aggregate into clumps and the apical plasma membrane to separate from the cell wall to form folds (Figure 1D). Together, these data indicate that LL37 binds directly to *A. fumigatus* and destroys cell wall integrity.

### LL37 Inhibits *A. fumigatus* Hyphae Growth and Adhesion *in vitro*

**Figure 2:**
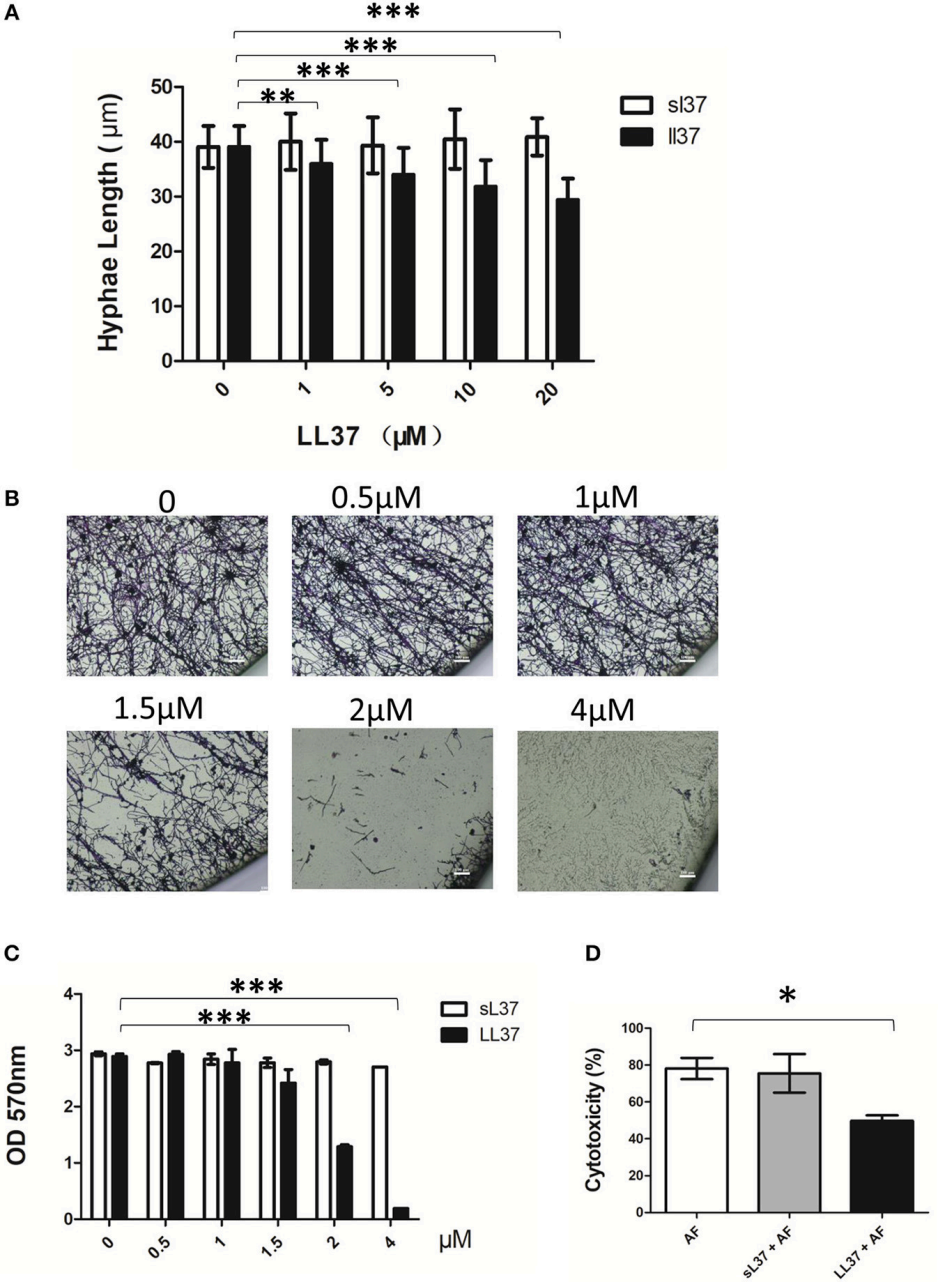
Effects of LL37 on *A. fumigatus* hyphal growth and adhesion. **(A)** *A. fumigatus* were incubated with different concentration of LL37 or sL37(0, 1, 5, 10, 20 μM) at 37°C for 12 h. Hyphal lengths were determined under a microscope. For each variable, at least 50 hyphal measurements were recorded. **(B,C)** *A. fumigatus* were incubated on polystyrene plates with different concentration of LL37 or sL37(0, 0.5, 1, 1.5, 2, 4 μM) at 37°C for 24 h. The wells were washed with PBS and stained with crystal violet. Then the wells were photographed under a microscope and the absorbance of decolorized solution at 570 nm after decoloring by ethanol was determined. **(D)** Alveolar epithelial cells (1 × 10^5^) were incubated with *A. fumigatus* (1 × 10^6^) in the presence or absence of 4 μM LL37 or sL37 at 37°C for 16 h. Cell viability was analyzed by the LDH released in the supernatant. The bars represent the mean values and the standard deviation. The data are representative of three independent experiments. *P < 0.05; **P < 0.01; ***P < 0.001. AF, *Aspergillus fumigatus*. sL37, scrambled-LL37.

To explore the effect of LL37 on the biological activity of *A. fumigatus*, we measured the hyphal length of *A. fumigatus* following 10 h of incubation with LL37 or sL37 at concentration gradients. Our results showed that hyphal growth was significantly inhibited by LL37 in a concentration-dependent manner (Figure 2A). Unexpectedly, we noticed that *A. fumigatus* were still viable even at high concentration of 20 μM LL37, which indicates that LL37 plays a role in inhibiting the growth of mycelium rather than sterilization. Furthermore, this inhibiting effect on hyphae growth resulted in impaired biofilm formation (Figure 2B). Crystal violet staining was applied to quantify the adherent biofilm density and it was shown that adherent biofilm formation was markedly attenuated even at a low dose of LL37 treatment (≤ 4 μM) (Figure 2C). To further exam whether or not LL37 impacts *A. fumigatus* invasion and destruction of epithelial cells, alveolar epithelial cells were incubated with *A. fumigatus* in the presence of LL37 or sL37 for 16 h, and then the secretion of LDH in the supernatant was measured to identify the cell death rate. The results indicated that LL37 attenuated *A. fumigatus* invasion and cytotoxicity on epithelial cells (Figure 2D). Overall, these findings suggest that LL37 inhibited *A. fumigatus* hyphae growth and adhesion *in vitro*.

### LL37 Inhibits *A. fumigatus*-Induced Pro-Inflammatory Cytokine Production in Macrophages

**Figure 3:**
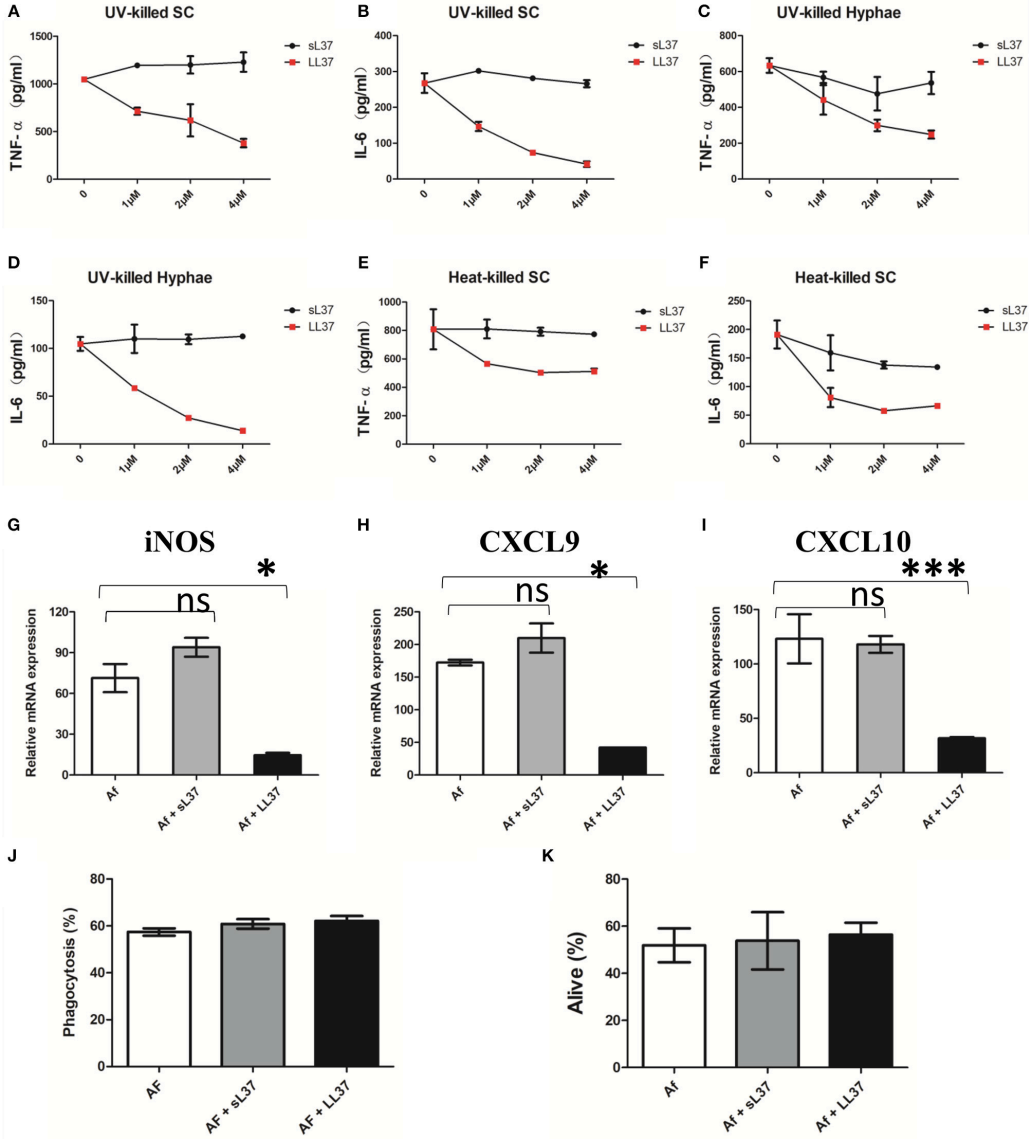
Effects of LL37 on the activation of *A. fumigatus*-infected macrophages. **(A–F)** Macrophages were incubated with ultraviolet-killed or heat-killed *A. fumigatus* swell conidia (MOI = 5:1) or hyphae (MOI = 1:1) in the presence of LL37 or sL37(0, 1, 2, 4 μM). TNF-α and IL-6 levels were determined at 16 h after stimulation. **(G–I)** Macrophages were incubated with ultraviolet-killed *A. fumigatus* swell conidia (MOI = 5:1) in the presence or absence of 4 μM LL37 or sL37. The relative mRNA expression of iNOS, CXCL9, CXCL10 were determined at 12 h after stimulation. **(J)** Macrophages were cocultured with FITC-labeled *A. fumigatus* conidia (MOI = 10) in the presence or absence of 4 μM LL37 or sL37 for 1 h. Then the macrophages were stained with anti-CD11b PerCP cy5.5 before being analyzed using a BD flow cytometer. The percentages of phagocytosis were calculated by the ratio of FITC^+^ macrophages to all macrophages. **(K)** Macrophages were incubated with viable *A. fumigatus* conidia (MOI = 1:5) in the presence or absence of 4 μM LL37 or sL37 for 6 h. The fungi were scraped off and diluted with PBS and then cultured on SDA plates to count colonies. The bars represent the mean values and standard errors of the means. The data are presented as the mean ± SD and representative of three independent experiments. *P < 0.05; ***P < 0.001. TNF-α, tumor necrosis factor-α. IL-6, interleukin-6. SC, swell conidia. UV, ultraviolet. iNOS, inducible nitric oxide synthase. CXCL, Chemokine (C-X-C motif) ligand. sL37, scrambled-LL37.

To assess the influence of LL37 on inflammatory activity in *A. fumigatus*-infected macrophage, BMDMs were stimulated with ulraviolet-killed or heat-killed *A. fumigatus* hyphae or swollen conidia in combination with different concentrations of LL37 or sL37 for 16 h, after which point, TNF-α and IL-6 in the supernatant were detected. In comparison with sL37 treatment, LL37 significantly reduced the *A. fumigatus*-induced TNF-α and IL-6 production in macrophages and it performed as a dose-dependent manner in ulreaviolet-killed groups (Figures 3A–F).

Meanwhile, we examined the transcriptional levels of macrophage polarization-related markers. Consistent with the reduced production of proinflammatory cytokines, LL37 also downregulated the mRNA expressions of iNOS, CXCL9 and CXCL10 (Figures 3G–I), which related to M1-type macrophages. However, the levels of M2-type macrophage—related markers, such as arginase and Fizz, were very low in all of groups following *A. fumigatus* stimulation (data not shown).

In addition, we wondered whether LL37 affected macrophage phagocytosis and killing capacity on *A. fumigatus* as it decreased inflammatory cytokines production, so we incubated *A. fumigatus* conidia with macrophages in the presence of LL37 or sL37. However, both LL37 and sL37 did not attenuate the phagocytosis or killing capacity of macrophages against *A. fumigatus* (Figures 3J,K). Together, those data suggest that LL37 inhibits *A. fumigatus*-induced proinflammatory cytokine production from macrophages and downregulates M1-type markers without affecting macrophage phagocytosis or killing capacity.

### LL37 Promotes Fungi Clearance and Alleviates Lung Pathological Injury in an *A. fumigatus*-Infected Mouse Model

**Figure 4:**
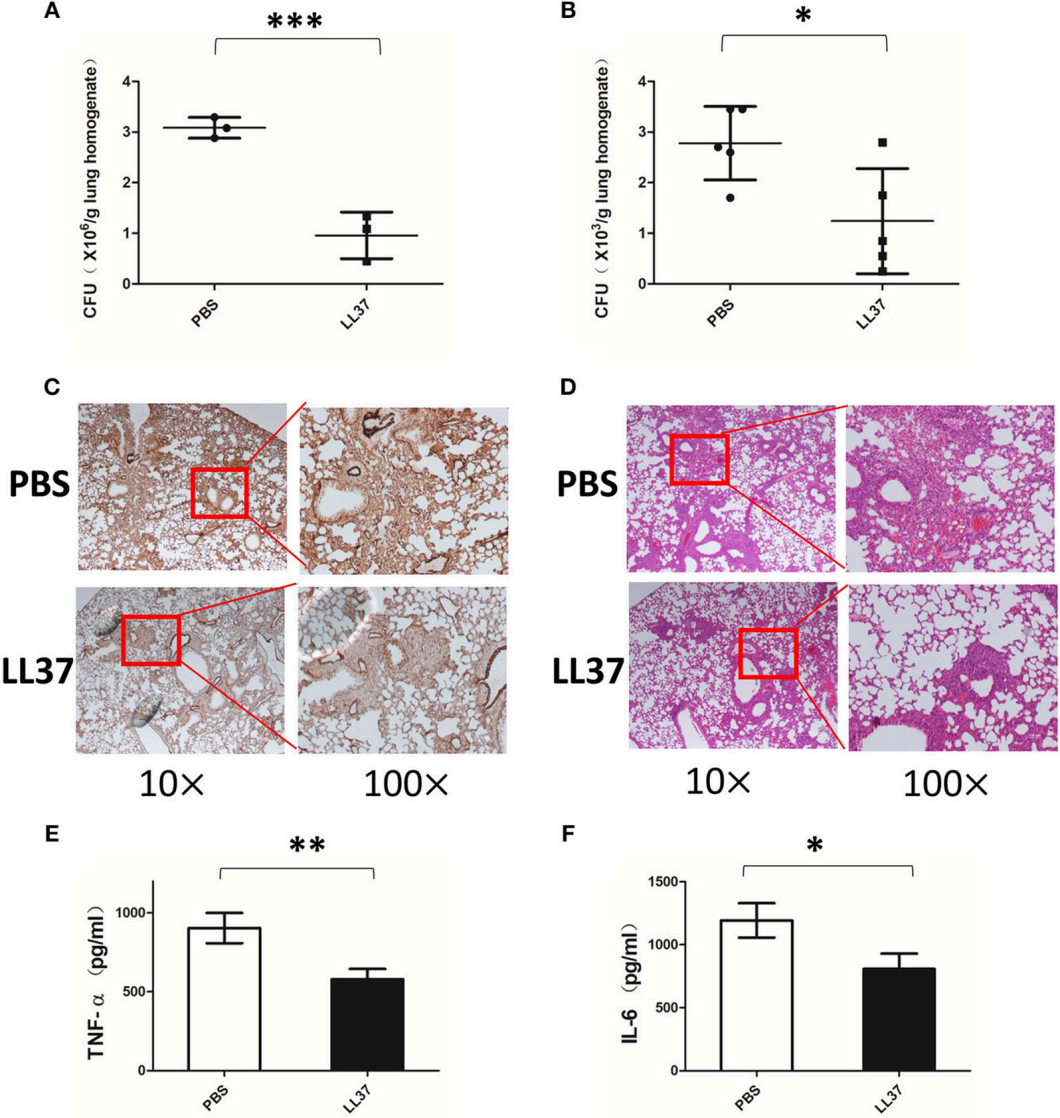
Exogenous LL37 acts on controlling *A. fumigatus* infection *in vivo*. C57 BL/6 mice were infected via intratracheal administration of 2 × 10^7^ *A. fumigatus* conidia, followed by supplement with 35 μL LL37(0.1 mg/mL) or PBS. **(A,B)** The fungal burden in lung tissues were determined at 2 days and 7 days after *A. fumigatus* infection, respectively. **(C,D)** Histopathological examination by PASM and HandE staining of mouse lungs were conducted at 7 days after *A. fumigatus* infection. The levels of TNF-α **(E)** and IL-6 **(F)** in lung homogenates were determined after 2 days of *A. fumigatus* infection. The data are representative of three independent experiments. The bars represent the mean values and standard deviation. *P < 0.05; **P < 0.01; ***P < 0.001. TNF-α, tumor necrosis factor-α. IL-6, interleukin-6. PBS, phosphate buffer saline.

The findings described above indicate that LL37 inhibits *A. fumigatus in vitro*, however, the role of LL37 in the *A. fumigatus* infection *in vivo* is not clear. To investigate this, we infected mice via intratracheal administration of 2 × 10^7^ *A. fumigatus* conidia followed by LL37 peptide solution or PBS instillation. As compared with mice in the control group, LL37-treated mice infected with *A. fumigatus* showed significantly lower fungi burdens in the lungs at both 2 and 7 days (Figures 4A,B; Figure S3 in Supplementary Material). Subsequently, we performed histological analysis of lung tissues from mice at 7 days after *A. fumigatus* infection. PASM staining was used to visualize the fungal distribution and revealed that the majority of fungi were confined around the trachea and bronchi in the lungs of LL37-treated mice, whereas *A. fumigatus* hyphae had spread into the alveolar tissues of the lungs of the control group (Figure 4C). Additionally, hematoxylin and eosin (H&E) staining of lung tissues revealed that a greater number of immune cells accumulated and the more severe phenomenon of congestion and structural damage happened in the mice of the control group vs. LL37-treated mice (Figure 4D). These results collectively indicated that LL37 prompted *A. fumigatus* clearance and controlled the hyphae invasion, which alleviated pathological damage.

### LL37 Inhibits *A. fumigatus*-Induced Pro-inflammatory Cytokines *in vivo*

Since LL37 inhibits *A. fumigatus*-induced pro-inflammatory cytokines *in vitro*, we sought to investigate whether the anti-inflammatory effects of LL37 were also available *in vivo*. The cytokine levels in lung homogenate were analyzed at 2 days after *A. fumigatus* infection. In line with our hypothesis, the release of TNF-α and IL-6 from the lungs of LL37-treated mice was significantly lower than that from the control group mice (Figures 4E,F).

### Endogenous LL37 Plays a Protective Role in *A. fumigatus* Infection

**Figure 5:**
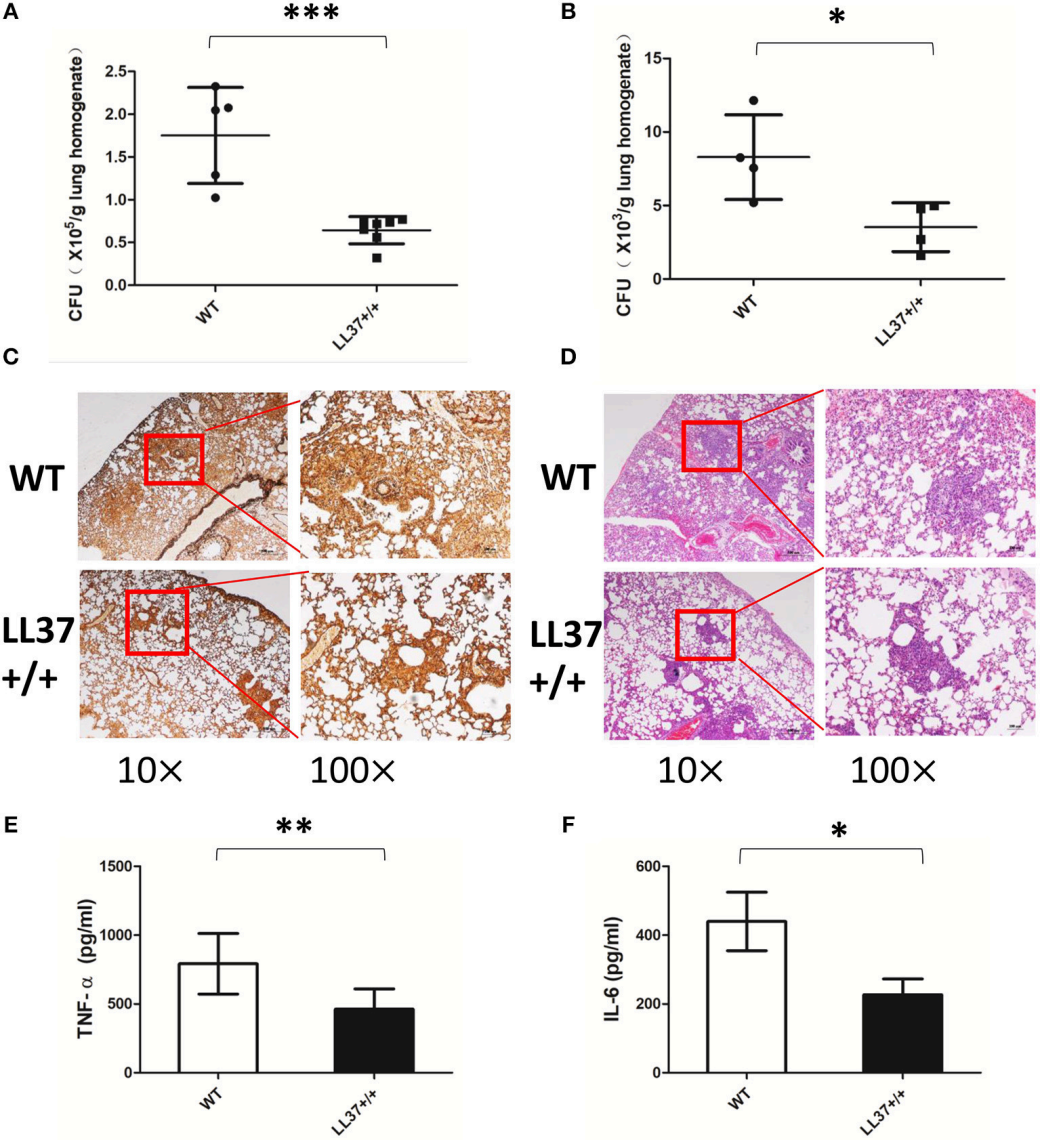
Endogenous LL37 promotes fungi eradication and alleviates pathological injury. Wild-type mice and LL37+/+ mice were infected by way of intratracheal administration of 2 × 10^7^ *A. fumigatus* conidia. After 2 days **(A)** and 7 days **(B)**, the fungal burden in the lungs were analyzed. **(C,D)** PASM and H&E staining outcomes of lungs tissues were analyzed after 7 days. TNF-α **(E)** and IL-6 **(F)** in lung homogenates were determined after 2 days. The bars represent the mean values and standard deviation. *P < 0.05; **P < 0.01; ***P < 0.001. WT, wild type. TNF-α, tumor necrosis factor-α. IL-6, interleukin-6.

Considering the complex interactions among molecules in the immune system, we constructed LL37 transgenic mice to explore whether endogenous LL37 played a protective role in *A. fumigatus* infection. We infected LL37+/+ and wild-type mice via intratracheal administration of 2 × 10^7^ *A. fumigatus* conidia. Both 2 days and 7 days after infection, the fungi load showed that the LL37+/+ mice had significantly smaller number of *A. fumigatus* in the lungs than did the wild-type mice (Figures 5A,B). At 7 days after infection, histopathological analysis was performed. PMSF staining revealed that wild-type mice had significantly more *A. fumigatus* invading lesions vs. the LL37+/+ mice (Figure 5C). H&E staining suggested that the lungs of wild-type mice were heavily infiltrated by inflammatory cells and the congestion and tissue destruction were more obvious in these mice vs. in the LL37+/+ mice (Figure 5D).

### Endogenous LL37 Alleviates Inflammation Caused by *A. fumigatus* Infection

**Figure 6:**
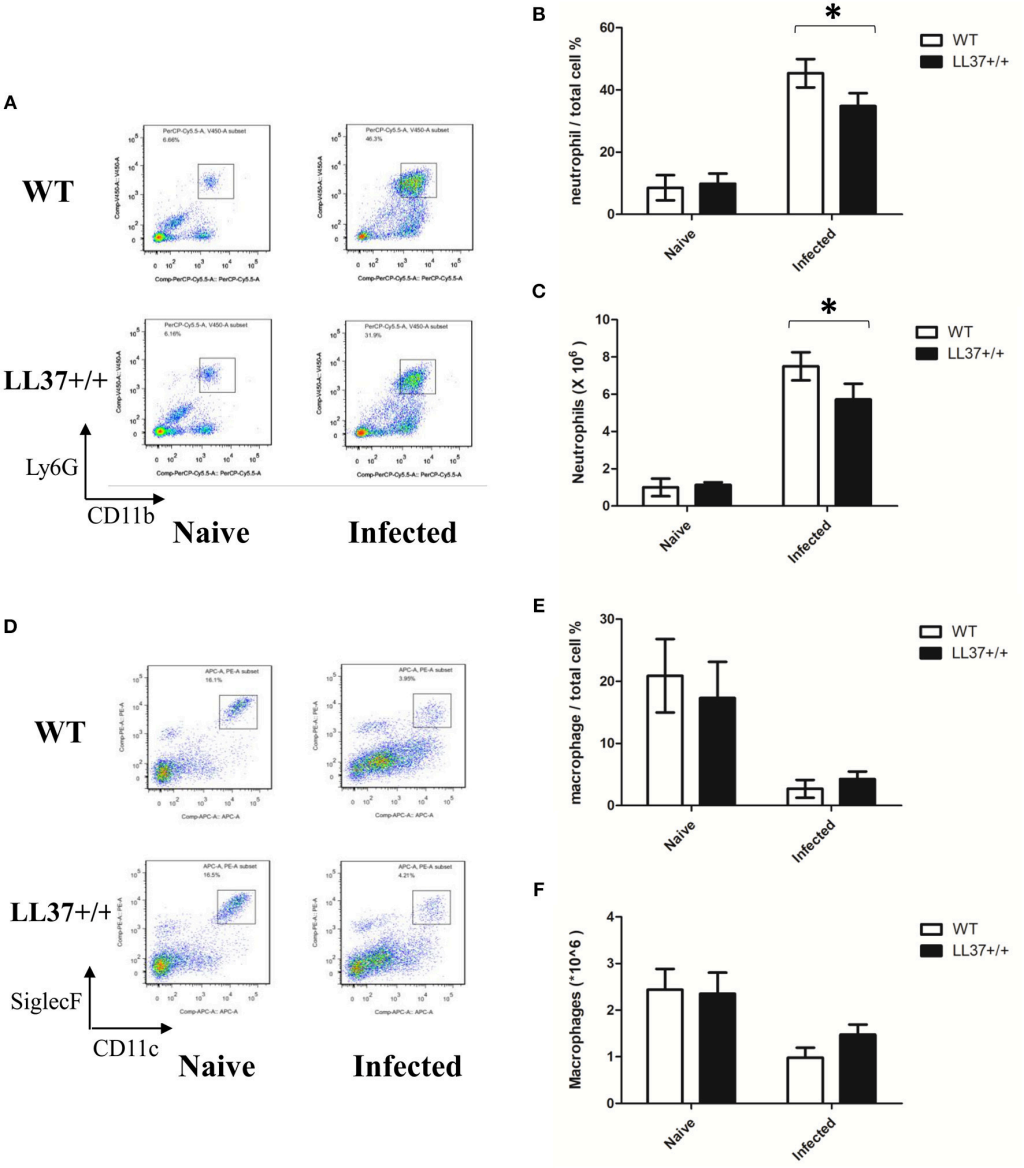
Effects of endogenous LL37 on neutrophils and macrophage infiltration in the lung infected by *A. fumigatus*. **(A–F)** Wild-type mice and LL37+/+ mice were infected via intratracheal administration of 2 × 10^7^ *A. fumigatus* conidia. After 2 days, the number of total cells in the lungs was counted, while the percentage of neutrophil and macrophage percentages were analyzed by flow cytometry. The data are representative of three independent experiments. The bars represent the mean values and standard deviation. *P < 0.05. WT, wild type.

To examine the effects of endogenous LL37 on the inflammatory response mediated by *A. fumigatus* infection, we analyzed inflammatory cells and inflammatory cytokines in mouse lung. The results showed that the lung tissues of LL37+/+ mice released less TNF-α and IL-6 than did those of wild-type mice after 2 days of infection (Figures 5E,F). Using flow cytometry, we additionally observed that LL37+/+ mice performed significantly reduced number and percentage of neutrophils in the lung vs. mice of the control group, who showed plenty of neutrophils (i.e., over 40% of total cells) recruited to the lung (Figures 6A–C). In contrast, there was little difference in the number or percentage of macrophages between LL37+/+ mice and control group mice, which both showed an obvious descent of macrophages after *A. fumigatus* infection (Figures 6D–F).

### LL37 Plays a Protective Role in an *A. fumigatus*-Infected Immunosuppressed Mouse Model

**Figure 7:**
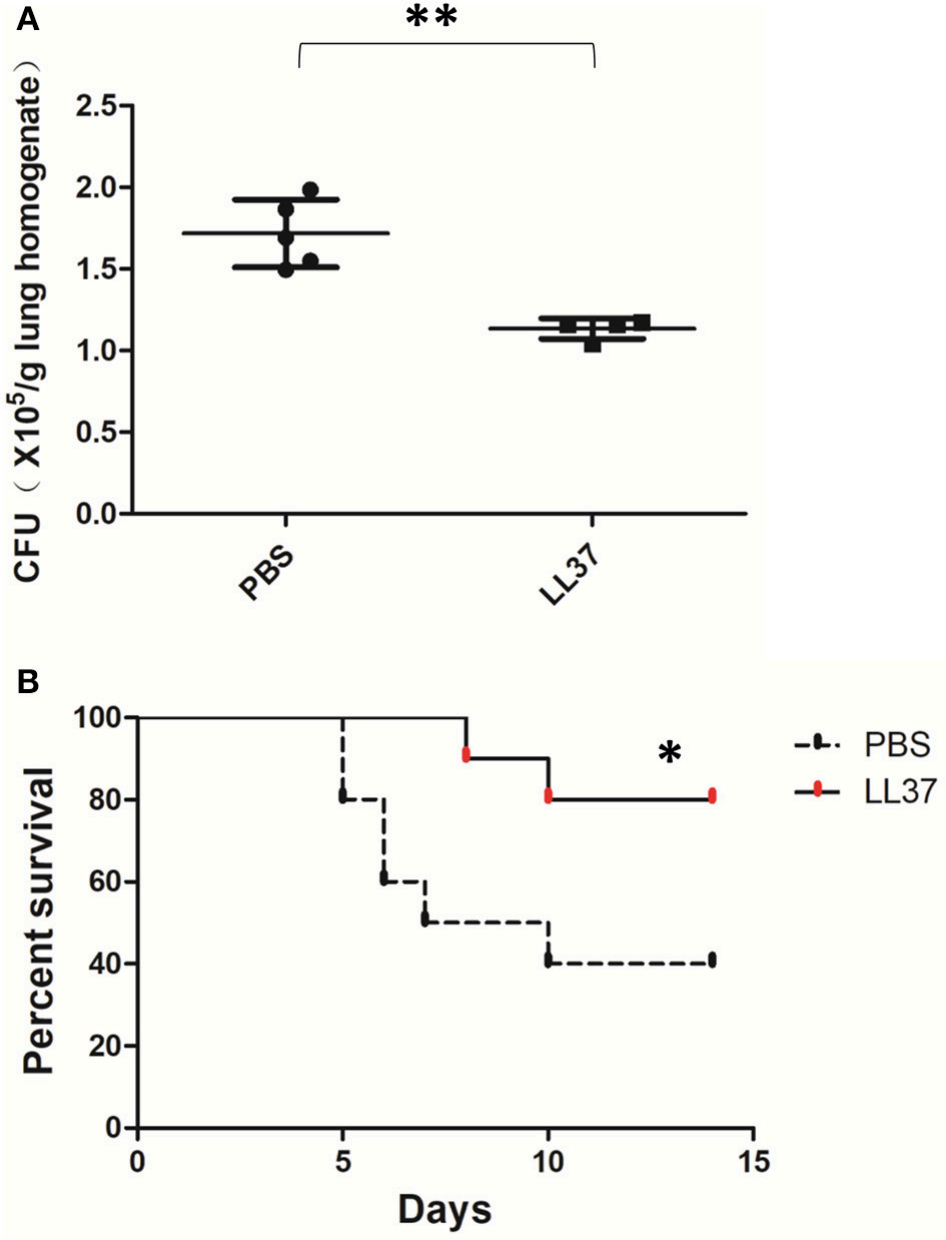
Effects of LL37 in an *A. fumigatus*-infected immunosuppressed mouse model. Corticosteroid-treated mice were infected via intratracheal administration of *A. fumigatus* conidia (2 × 10^6^ for fungal burden, 5 × 10^6^ for survival), followed by supplement of 35 μL LL37 (0.1 mg/mL) or PBS. **(A)** The fungal burden in lung tissues was determined after 2 days of *A. fumigatus* infection. **(B)** Survival was monitored for 2 weeks. **P < 0.05. *P < 0.05.

Considering the data above, we still wondered whether the protective effects of LL37 *in vivo* were directly implemented on the fungus or indirectly enacted by reducing the associated inflammation. Further, we examined the role of LL37 during infections in corticosteroid-treated mice, which imitated the effects of immunosuppression. Under these conditions, LL37-treated mice infected with *A. fumigatus* for 2 days showed obviously lower fungi burdens in the lungs as compared with mice in the control group (Figure 7A). Furthermore, the survival rate curve suggested that LL37-treated mice were significantly less susceptible to *A. fumigatus* infection vs. control group ones (Figure 7B). These results indicated that the direct inhibition of LL37 on *A. fumigatus* contributed to its protective role *in vivo*.

## Discussion

LL37, as a host defense peptide, is known to have a broad antibacterial spectrum, including against both Gram-negative and Gram-positive bacteria (10). Meanwhile, the fungicidal activity of LL37 against *Candida albicans* (19–21) and CAMP from other species against *Cryptococcus neoformans* have also been reported recently (22). Therefore, our interest was piqued regarding exploring the biological effects of human-derived LL37 on *A. fumigatus*, which is the most important pathogen of aspergillosis. The current study provided evidence that LL37 bind to *A. fumigatus* and destroyed its architecture, resulting in inhibiting hyphae growth and adhesion *in vitro*. Meanwhile, LL37 prevented *A. fumigatus*-induced aggressive macrophages activation. Further, both exogenous and endogenous LL37 prompted the elimination and prevented the invasion of fungi *in vivo*, and also reduced pathological damage and inflammation.

As mentioned above, the bactericidal activity of LL37 has been described as the model of peptide-membrane interaction. In contrast to the structure and composition of the bacterial cell membrane, fungi show thicker cell walls and different polysaccharides that contain more zwitterionic phospholipids and sterols (23, 24). Thus, LL37 may exert different mechanism against fungi. Regarding another common fungal pathogen, *C. albicans*, studies have reported that LL37 performs its fungicidal effect through membrane permeability as well as by affecting fungal structural integrity and altering cell wall composition (20, 21, 25). Our results suggested that LL37 could bind to the surface of *A. fumigatus*, which was confirmed by fluorescence confocal microscopy. Further, we observed that LL37 disrupted the cell wall structure and caused the cytoplasmic membrane at the apical side to separate from the cell wall to form a wavy structure under the electron microscope. However, the mechanism of LL37 acting on *A. fumigatus* directly via activating glycanase or forming pores on cell wall/membrane, or indirectly via triggering stress response pathways, remains to be further studied.

Our experiments additionally suggested LL37 inhibited mycelial growth in a dose-dependent manner, but we noted that *A. fumigatus* could still grow into hyphae that were just a little shorter than the normal at high concentration of LL37 (e.g., 20 μM). This is consistent with the findings of previous literature reports that indicated the minimum inhibitory concentrations of five CAMP peptides derived from cattle, sheep and pigs against *Candida* and *Cryptococcus* are in the range of 0.5–32 μM, while filamentous fungi, such as *Aspergillus*, are less susceptible to these peptides (22).

Interestingly, while the mycelial growth of *A. fumigatus* was almost unaffected at low concentrations of LL37 (e.g., 4 μM), its adhesion ability in comparison was significantly diminished. Therefore, the primary function of LL37 on *A. fumigatus* may tend to inhibit invasion rather than act in a fungicidal manner, which is not the same effects as on bacteria. This is in line with our *in vivo* experiments wherein pathological foci showed that *A. fumigatus* in the lungs of LL37 transgenic mice was mainly confined around trachea and bronchi but disseminated to the alveolar and pulmonary vessels in wild-type mice. As with *A. fumigatus*, LL37 significantly inhibited the adhesion of *C. albicans*, which is related to the elevation of β-1,3-exoglucanase activity (26). However, further research is needed to investigate the mechanism(s) how LL37 inhibits adhesion and invasion of *A. fumigatus*.

Other studies have demonstrated that LL37 inhibits inflammation by neutralizing LPS and inhibiting TLR4 activation in bacterial infections (27–30). The present investigation suggested that TNF-α and IL-6 levels from *A. fumigatus*-stimulated macrophages incubating with LL37 also decreased. Given that cell wall components often serve as PAMPs to trigger pro-inflammatory responses in innate cells such as macrophages, there is reason to speculate that impaired cell wall integrity by LL37 accounts, at least partly, for reduced production of pro-inflammatory cytokines. However, there was no change of TNF-α and IL-6 production in LL37 treated-macrophages in the absence of infection vs. in untreated group, neither in macrophages infected by Aspergillus pre-treated with LL37 or sL37 for an hour vs. by untreated *A. fumigatus* (data not shown). In order to distinguish the direct fungistatic and immunomodulatory effects of LL37, we treated *A. fumigatus* by ultraviolet inactivation and heat inactivation treatment, followed by stimulation. The results showed that LL37 could still significantly inhibit the production of TNF-α and IL-6 both in ultraviolet- and heat- killed groups, which indicated LL37 mainly acted by immunomodulation rather than via direct fungistatic effects to reduce inflammation in the situation we set.

To explore how LL37 regulates inflammation in *A. fumigatus*-infected macrophages, we examined the expression of macrophage polarization-related makers and found that LL37 downregulated M1 type markers, such as iNOS, CXCL9 and CXCL10. It has been reported that LL37 can directly induce macrophages to differentiate into M1 type with proinflammatory effects (31). Therefore, we can assume that the immunomodulatory effect of LL37 is closely related with the type of pathogens.

Inflammatory response is a reasonable manifestation of a host's defense against invasive pathogens; however, excessive inflammation can cause damage to the host tissues and even adversely affect the pathogen clearance (6, 32, 33). As seen in our animal experiments, the percentage of neutrophils in the lungs of wild-type mice infected with *A. fumigatus* was as high as 40%, which resulted in severe edema, congestion and destruction of lung tissue. In contrast, the lungs of LL37+/+ mice experienced a relatively lesser degree of inflammatory cells infiltration, more effective pathogen clearance, and reduced lung damage. Although *A. fumigatus* is clinically susceptible to immunosuppressed patients, it also occurs in patients with high levels of inflammation such as chronic granulomatous disease or cystic fibrosis, who show host damage and impaired fungi eradication (34, 35). Therefore, for the treatment of aspergillosis, in addition to considering the killing of fungi, the promotion of protective immune response regulation is also crucial.

Furthermore, our data demonstrated that LL37 still reduced the degree of susceptibility to *A. fumigatus* in immunosuppressive mouse models and suggested that the direct inhibition of LL37 on *A. fumigatus* contributed to its protective role *in vivo*. We suppose that LL37 significantly inhibits mycelium adhesion and invasion *in vivo* as shown in the *in vitro* experiments.

However, *in vitro* experiments cannot explain the role of LL37 in different microenvironments *in vivo* (36, 37). Even if exogenously synthesized LL37 was used in mouse experiments, it could not fully reflect the complex interactions that may occur in the innate immune system. In this study, we established LL37 transgenic mice by microinjection of linearized plasmids expressing hCAP18/LL37 into fertilized eggs and demonstrated that endogenous LL37 had a protective effect against *A. fumigatus* infection. As this transgenic mouse overexpressed LL37 systemically, further experiments are needed to identify the alveolar epithelium or neutrophils where the LL37 derived from that play a major role.

It is worth noting that a recent study reported that LL37 promoted the growth of *A. fumigatus* (38). However, in addition to the differences between fungal strains we used (Af293 vs. ATCC strain), the authors were mainly concerned about the effects of much lower levels of LL37 (i.e., 5 μg/ml, approximately 1 μM) than us at mostly higher than 1 μM. In growth experiments, their lower concentrations of LL37 (7.8–1.9 μg/ml) were more conducive to *A. fumigatus* growth, while we focused on the inhibition of hyphal length and adhesion by larger doses of LL37. As the authors explained in the discussion, a low level of stress may activate compensatory growth pathways in *A. fumigatus*. Therefore, it is necessary to carry out further rigorous experiments to determine the LL37 concentration threshold that causes completely opposite effects before LL37 can be used to treat *A. fumigatus* infection in the future.

In conclusion, LL37 directly inhibits *A. fumigatus* mycelial growth and its adhesion and invasion ability. Meanwhile, there is beneficial immunomodulatory effect—LL37 attenuates the *A. fumigatus*-induced excessive inflammatory response. Taken together, it can be surmised that LL37 exerts a dual protective effect in the treatment of *A. fumigatus* infection and it and its analogs may be potential drug components for use against *A. fumigatus* infection.

## Author Contributions

X-LL designed, performed, and analyzed the experiments and wrote the manuscript. J-XL designed, performed and analyzed the experiments. J-FX and X-MJ designed, analyzed and provided overall guidance for the experiments, wrote and revised the manuscript. H-RH helped with the experiments and interpretation of the data. J-LD, R-XD, R-JT, LY, and JH contributed to the experiments.

### Conflict of Interest Statement

The authors declare that the research was conducted in the absence of any commercial or financial relationships that could be construed as a potential conflict of interest.
