# Supplementary material for: LL37 Inhibits Aspergillus fumigatus Infection via Directly Binding to the Fungus and Preventing Excessive Inflammation
Source: Front Immunol. 2019 Feb 20;10:283. doi: 10.3389/fimmu.2019.00283 (PMC6391356; doi:10.3389/fimmu.2019.00283)
Supplement: Supplementary file 1 [file Table_1.DOCX]

Supplementary Material

LL37 inhibits Aspergillus fumigatus infection via directly binding to the mycelium and preventing excessive inflammation

Xiao-Li Luo^#^, Jian-Xiong Li^#^, Hua-Rong Huang, Jie-Lin duan, Ruo-Xuan Dai, Ru-Jia Tao, Ling Yang, Jia-yun Hou, Xin-Ming Jia*, Jin-Fu Xu*

*** Correspondence:** Jin-Fu Xu: [jfxucn@gmail.com](mailto:jfxucn@gmail.com); or Xin-Ming Jia: jiaxm@tongji.edu.cn.

# Supplementary Data

Generation and identification of LL37 transgenic mice (LL37+/+)

To create LL37+/+ mice, we first constructed the LL37-expressing plasmid. Human LL37 complementary DNA (NCBI: NM_004345.4) was inserted into an IRES vector with a GFP expression sequence, which was driven by a constitutively active EF-1ɑ promoter. Then, the pIRES human LL37 expression plasmid was microinjected into fertilized eggs obtained from FVB background mice. Finally, LL37+/+ mice were generated by implanting the processed fertilized eggs into FVB females.

To identify the LL37+/+ mice, genotyping polymerase chain reaction (PCR) was performed to analyze LL37 gene expression on tails that were obtained from mice aged between 3- and 4-weeks old. PCR was performed using the following primers: LL37 F: 5’- AGCAGTCACCAGAGGATTGT-3’ and LL37 R: 5’- GGCACACACTAGGACTCTGT-3’. The PCR products produced by LL37+/+ mice were 250 bp long. Next, Western blotting was used to detect LL37 protein expression following extraction from lung tissues. Wild-type (FVB) mice were used as controls.

# Supplementary Figures and Tables

## Supplementary Figure 1


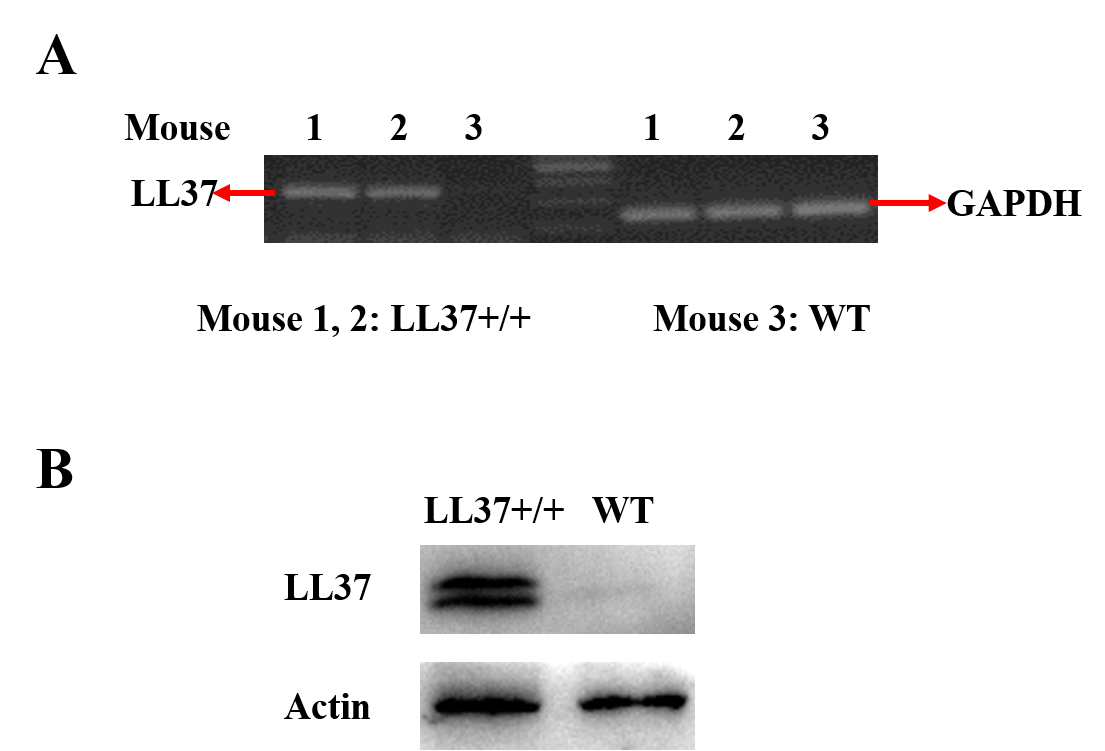


**Supplementary Figure 1.** Identification of LL37+/+ mice from the perspective of DNA and protein expression. A. PCR was performed to analyze LL37 gene expression on mice tails. B. Western blotting was used to detect LL37 protein expression of lung tissues.

## Supplementary Figure 2

**
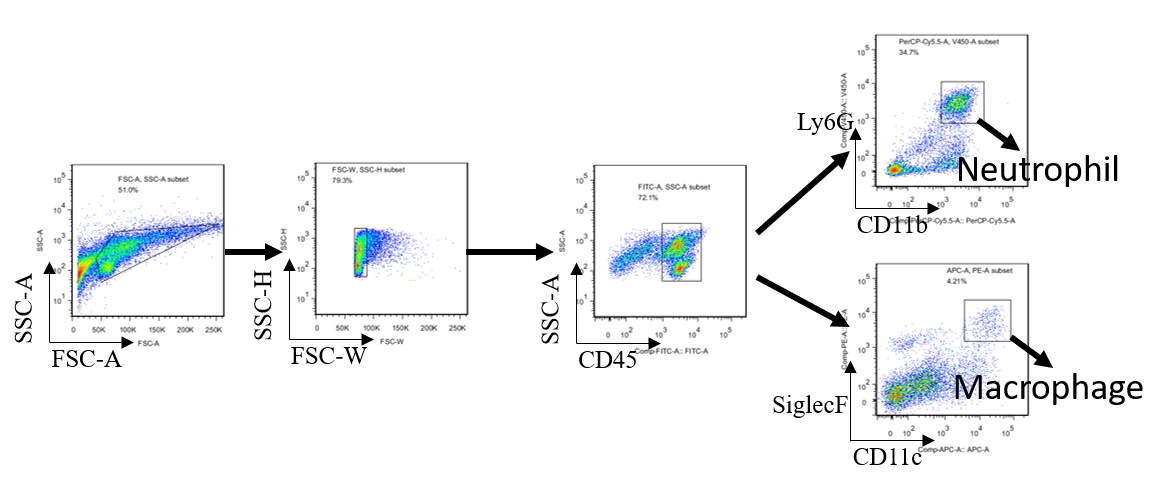
Supplementary Figure 2.** Determining neutrophils and macrophages infiltration in the lung infected by *A. fumigatus*. Gating strategy for flow assay of alveolar macrophage (CD11c ^+^ SiglecF ^+^), and neutrophil (CD11b ^+^ Ly6G ^+^) counts in lungs of WT and LL37+/+ mice at 2 days after intratracheal administration of 2×10^7^ A. fumigatus conidia.

## Supplementary Figure 3


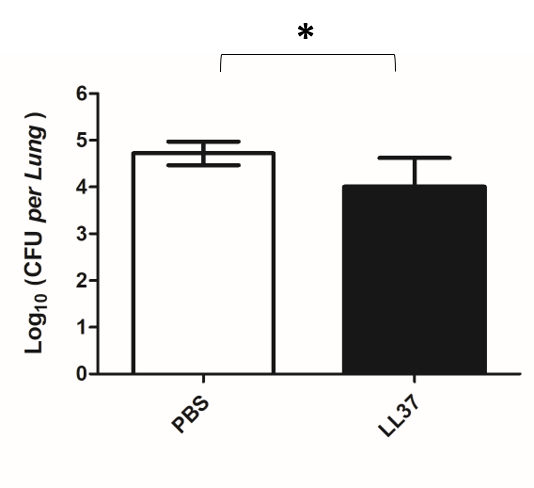
**Supplementary Figure 3.** Fungal burden in the lungs of mice infected with *A. fumigatus* and treated with LL37 or PBS using real-time PCR. Mice were killed at 2 days after 2×10^7^ *A. fumigatus* infection and lungs were dissected carefully. DNA of the lung sample was extracted with the E.Z.N.A. fungal DNA kit (Omega Bio-Tek, Norcross, GA). Six-point standard curves were calculated using serial dilutions of AF293 strain genomic DNA. A Taqman probe qPCR assay was used to quantify fungal DNA as previously described (Morton et al., 2011). Total fungal DNA was calculated comparing the threshold cycle (C_T_) value with the appropriate standard curve and normalized to input DNA concentration.
